# Genomic Regions Associated with Feed Efficiency Indicator Traits in an Experimental Nellore Cattle Population

**DOI:** 10.1371/journal.pone.0164390

**Published:** 2016-10-19

**Authors:** Bianca Ferreira Olivieri, Maria Eugênia Zerlotti Mercadante, Joslaine Noely dos Santos Gonçalves Cyrillo, Renata Helena Branco, Sarah Figueiredo Martins Bonilha, Lucia Galvão de Albuquerque, Rafael Medeiros de Oliveira Silva, Fernando Baldi

**Affiliations:** 1 Universidade Estadual Paulista, Faculdade de Ciências Agrárias e Veterinárias, Departamento de Zootecnia, Via de acesso Prof. Paulo Donato Castellane, s/no, CEP 14884-900 Jaboticabal, SP, Brazil; 2 Instituto de Zootecnia, Centro Avançado de Pesquisa Tecnológica do Agronegócio de Bovinos de Corte, Rodovia Carlos Tonanni, km 94, CEP 14.174-000, Sertãozinho, SP, Brazil; Universitat de Lleida, SPAIN

## Abstract

The objective of this study was to identify genomic regions and metabolic pathways associated with dry matter intake, average daily gain, feed efficiency and residual feed intake in an experimental Nellore cattle population. The high-density SNP chip (Illumina High-Density Bovine BeadChip, 777k) was used to genotype the animals. The SNP markers effects and their variances were estimated using the single-step genome wide association method. The (co)variance components were estimated by Bayesian inference. The chromosome segments that are responsible for more than 1.0% of additive genetic variance were selected to explore and determine possible quantitative trait loci. The bovine genome Map Viewer was used to identify genes. In total, 51 genomic regions were identified for all analyzed traits. The heritability estimated for feed efficiency was low magnitude (0.13±0.06). For average daily gain, dry matter intake and residual feed intake, heritability was moderate to high (0.43±0.05; 0.47±0.05, 0.18±0.05, respectively). A total of 8, 17, 14 and 12 windows that are responsible for more than 1% of the additive genetic variance for dry matter intake, average daily gain, feed efficiency and residual feed intake, respectively, were identified. Candidate genes *GOLIM4*, *RFX6*, *CACNG7*, *CACNG6*, *CAPN8*, *CAPN2*, *AKT2*, *GPRC6A*, and *GPR45* were associated with feed efficiency traits. It was expected that the response to selection would be higher for residual feed intake than for feed efficiency. Genomic regions harboring possible QTL for feed efficiency indicator traits were identified. Candidate genes identified are involved in energy use, metabolism protein, ion transport, transmembrane transport, the olfactory system, the immune system, secretion and cellular activity. The identification of these regions and their respective candidate genes should contribute to the formation of a genetic basis in Nellore cattle for feed efficiency indicator traits, and these results would support the selection for these traits.

## Introduction

Costs associated with animal feeding can be up to 50% of the total cost in beef cattle production systems, and there is growing interest concern in adopting strategies to reduce these costs [1]. So improving feed efficiency would increase both sustainability and profitability in the beef cattle industry. Selection for feed efficiency indicator traits in beef cattle can reduce production cost, decrease use of natural resources, and reduce impacts on the environment, optimizing production efficiency [2].

Feed efficiency (Kg gain/ Kg feed intake) showed a moderate positive correlation with weight gain and mature weight [3]. Thus, selection to improve feed efficiency could increase mature weight and increase energy maintenance requirements [4]. Also, residual feed intake (RFI), as proposed by Koch et al. [5], defined as the difference between actual feed intake and the feed intake required to meet maintenance requirements and growth, offers advantages over G:F since there is no correlation with performance traits [2, 6, 7, 8, 9].

Heritability estimates for RFI have been reported as moderate by many authors, ranging from 0.30 to 0.45 [3, 10, 11, 12, 13], suggesting that this trait can be improved by selection. However, RFI is difficult and expensive to measure, which often limits its implementation a selection criteria in beef cattle breeding programs. Recently, genome wide association studies (GWAS), using a high-density genotyping array, have been applied aiming to discover genomic regions associated with feed efficiency traits [11, 14, 15, 16]. It is important to highlight that most of these studies have been implemented with taurine breeds (*Bos taurus*), and there are few studies for indicine breeds (*Bos indicus*) under tropical conditions.

Therefore, in order to identify genomic regions associated with feed efficiency indicator traits, as well as to elucidate the genetic basis of them, it is important to encourage genomic studies with zebu animals, since zebu breeds are prevalent in herds under tropical and subtropical conditions. The objective of this study was to identify genomic regions and metabolic pathways associated with dry matter intake (DMI), average daily gain (ADG), feed efficiency (G:F) and residual feed intake (RFI) in an experimental Nellore cattle population.

## Material and Methods

### Data

This study was approved by ethics committee of the Faculty of Agrarian and Veterinary Sciences, Sao Paulo State University (UNESP).

The data set used in this study was provided by the APTA Beef Cattle Center—Institute of Animal Science (IZ), Sertãozinho, São Paulo, Brazil. Phenotypic information is animals born from 2004 to 2012. These animals belong to three experimental lines of Nellore cattle, which have been selected since 1978 for yearling weight: selection line (NeS) is a closed herd selected for higher yearling weight; traditional line (NeT) is submitted to the same selection criterion as NeS but, eventually, receives animals from NeS; and a control line (NeC) selected for average of yearling weight [17]. The analyzed data was obtained by feed efficiency tests performed from 2005 to 2013, consisting of 541 males and 355 females. Some of these animals (n = 683) were restricted to individual troughs, which offered daily feed and refusal was controlled, while the others (n = 213) were held in two collective pens equipped with the GrowSafe^®^ feeding system.

After weaning, animals were kept in the test for during 83±15 days, preceded by 28 days of adaptation, for evaluation the feed intake and average daily gain (ADG). Animals were weighed every 14 days after 12 hours of fasting. The diet was formulated with 67% of total digestible nutrients (TDN) and 13% of crude protein (CP), allowing ADG of 1.0 kg/day.

### Traits

In order to ensure *ad libitum* feed intake the food supply was adjusted daily, allowing refusals varying from 5–10% of offer. The following feed intake data was not considered in the analyses: for days when animals were handled outside of the facilities for a number hours, during failure (GrowSafe) and when no refusals were found. Diet dry matter percentage was determined from weekly samples of offer and refusals. The ADG in each test was considered as the linear regression coefficient of body weight (BW) on days in test (DIT):

$$y_{i}=\alpha +\beta *DIT_{i}+\varepsilon_{i}$$

where, *y*_*i*_ = BW in *i*^*th*^ observation; *α* = intercept of regression equation corresponding to the initial BW; *β* = linear regression coefficient corresponding to ADG; *DIT*_*i*_ = days in test for *i*^*th*^ observation; and *ε*_*i*_ = random error associated with each observation. Metabolic weight (BW^0.75^) was calculated as: $BW^{0.75}=\left[\;\alpha +\;\beta *\left(\frac{DIT_{i}}{2}\right)\right]{\;}^{0.75}$, with *α* and *β* assuming the values obtained by the equation described above.

Feed efficiency (G:F) was calculated as the ratio of ADG to DMI. The residual feed intake (RFI) was considered as error of linear regression equation of dry matter intake on average daily gain and metabolic weight within each contemporary group (CG: sex, year of birth, and pen), as shown below:

$$DMI=\beta T*TG+\beta TA*TG*ADG+\beta TB*TG*BW^{0.75}+\varepsilon$$

where, *βT*, *βTA*, and *βTB* are regression coefficients of classificatory variable test group (*TG*) and of interactions between *TG* and covariates *ADG* and *BW*^*0*.*75*^, respectively; and *ε* is RFI. The descriptive statistics for DMI, ADG, G:F and RFI are presented in Table 1.

**Table 1:** Descriptive statistics for dry matter intake (DMI), average daily gain (ADG), feed efficiency (G:F) and residual feed intake (RFI).

Trait	N^1^	Mean	SD^2^	Minimum	Maximum
ADG (kg BW /day)	896	0.996	0.26	0.18	1.71
DMI (kg DM / day)	896	6.70	1.24	3.65	19.10
G:F (kg BW / kg DM)	896	0.15	0.03	0.05	0.27
RFI (Kg DM / day)	896	0.0015	0.60	-2.28	4.96

^1^ N: the total number of phenotyped animals.

^2^ SD: standard deviation.

### DNA extraction

The extraction of DNA from blood samples was performed using the DNeasy Blood & Tissue Kit (Qiagen). The DNA purification was performed using a column containing silica fragments (column purification). Firstly, DNA binds to the membrane of the extraction column and then it was washed until it has high purity. At the end of the protocol, the DNA was eluted with buffer AE (blood). The amount of scanning and quality of the material obtained was taken with the use of a spectrophotometer apparatus (NanoDrop 1000, Thermo Scientific, USA, 2008). Quality was measured by absorption ratio A260 / A280. A ratio of less than 1.8 suggests contamination from protein, and more than 2.0 suggests RNA contamination.

### Genotyped Animals

Animals were genotyped using the high-density SNP chip (Illumina High-Density BovineBeadChip 777,000 SNP). Markers with minor allele frequency (MAF) and call rate higher than 5% and 95%, respectively, were considered, as well as samples with a call rate higher than 93%. After quality control of markers, 438,874 SNPs for 689 animals were available.

### Quantitative genetic analyses

The contemporary groups (CGs) included animals born on the same farm in the same year, and from the same management group as yearlings. The CGs with fewer than 3 records were eliminated from the analyses to maintain variability in the CGs. Records exceeding 3 standard deviations above or below the mean of each CG were excluded, to avoid the inclusion of possible measurement error or outliers. The model included the random additive animal effect, the fixed effects of CG, calving month, age of animal at beginning of the test (linear effect), and the dam age as co-variable (linear and quadratic effect). The (co)variance components were estimated using the single step genomic BLUP (ssGBLUP), under Bayesian inference [18]. The ssGBLUP is a modified version of the animal model (BLUP) with additive relationship matrix **A**^-1^ replaced by **H**^-1^ [19]:

$$H^{-1}=A^{-1}+\;\left[\begin{array}{cc} 0 & 0\\ 0 & G^{-1}-\;A_{22}^{-1} \end{array}\right]$$

where **A**_22_ is a numerator relationship matrix for genotyped animals and ***G*** is the genomic relationship matrix created as described by VanRaden et al. (2009) [20]:

$$G=ZDZ^{\prime }q$$

where *Z* is the gene matrix containing allele frequency adjustment; *D* is the matrix that have the SNP weight (initially *D* = ***I***); and, q is a weighting / standardization factor. According to Vitezica et al. (2011) [21], such factors can be obtained by ensuring that the ***G*** average diagonal is next to **A**_22_ The model can be represented by the following matrix equation:

y=Xβ+Za+e

where *y* is the observations vector; *β* is the vector of fixed effects; *a* is the additive direct vector; *X* is known as incidence matrix; *Z* is the incidence genetic random effects additive direct matrix (the *β* vector associated with the *y* vector); *e* is the residual effect vector. The *priori* distributions of vectors *y*, *a* and e were given by:

y~MVN(Xβ+Za)

a│G~MVN(0,H⊗G)

e│R~MVN(0,I⊗R)

where ***H*** is the relationship coefficients matrix among animals obtained from the single-step analyzes (*single-step)*; *R* is the residual variance matrix; *I* is the identity matrix; *G* is the genetic additive variance matrix and ⊗ is the Kronecker product. An inverted qui-square distribution was used for the prior values of the direct and residual genetic variances. A uniform distribution was used for the *priori* for the fixed effects a uniform distribution. A total of 3,342 animals were considered in the pedigree file. Analyses were performed using GIBBS2F90 [19, 22]. The a posteriori estimates were obtained using the application POSTGIBBSF90 [22].

The analyses were originated from chain lengths of 1,000,000 interactions, where the first 200,000 interactions were discarded. For parameter estimation, the samples were stored at each 100 cycles, building samples with 80,000 information. The data convergence was verified with the interactions versus evaluation graphic of sampled values and using the criteria proposed by Geweke; Heidelberger, Welch; and Raftery, Lewis [23–25] through analysis package Bayesian Output Analysis (BOA) in the software R 2.9.0 (The R Development Core Team, 2009).

### Genome-wide association analysis

Genome-wide association analysis for each trait was performed using the single-step GWAS (ssGWAS) methodology [26]. The same linear animal model used to estimate the (co)variance components was applied. The effects were decomposed in genotyped (*a*_*g*_) and ungenotyped (*a*_*n*_) animals, as describe by Wang et al. [26], considering the effect of genotyped animals as:

$$a_{g}=Zu,$$

where ***Z*** is a matrix that relates genotypes of each locus and ***u*** is a vector of marker effects, and the variance of animal effects was assumed as:

$$var\left(a_{g}\right)=var\left(Zu\right)=ZDZ^{\prime }\sigma_{u}^{2}=G^{*}\sigma_{a}^{2},$$

where *D* is a diagonal matrix of weights for variances of markers (*D* = ***I*** for GBLUP), σ^2^_u_ is the genetic additive variance captured by each SNP marker when no weights are present, and G* is the weighted genomic relationship matrix.

The ratio of covariance of genetic effects (*a*_*g*_) and SNPs (*u*) is:

$$var\;\left[\begin{array}{c} a_{g}\\ u \end{array}\right]=\;\left[\begin{array}{cc} ZDZ^{\prime } & ZD^{\prime }\\ DZ^{\prime } & D \end{array}\right]\sigma_{u}^{2},$$

sequentially:

$$G^{*}=\frac{var\left(a_{g}\right)}{\sigma_{a}^{2}}=\frac{var\left(Zu\right)}{\sigma_{a}^{2}}=ZDZ^{\prime }\lambda$$

where *λ* is a variance ratio or a normalizing constant. According to VanRaden et al. [20],

$$\lambda =\frac{\sigma_{u}^{2}}{\sigma_{a}^{2}}=\frac{1}{\sum_{i=1}^{M}2p_{i}\left(1-p_{i}\right)},$$

where *M* is the number of SNP and *p*_*i*_ is the allele frequency of the second allele in the *i*^*th*^ SNP. According to Stranden e Garrick [27], the markers effects can be described by:

$$û=\frac{\sigma_{u}^{2}}{\sigma_{a}^{2}}DZ^{\prime }G^{*-1}{\hat{a}}_{g}=DZ^{\prime }{\left[ZDZ^{\prime }\right]}^{-1}{\hat{a}}_{g},$$

The estimated SNP effects can be used to estimate the variance of each individual SNP effect [28] and apply a different weighting for each marker, such as:

$${\hat{\sigma }}_{û,i}^{2}={\hat{u}}_{i}^{2}2p_{i}\left(1-p_{i}\right)$$

The following iterative process described by Wang et al. [26] was used considering D to estimate the SNP effects:

1. *D* = ***I***,
2. To calculate the matrix ***G*** = *ZDZ’q*
3. To calculate GEBVs for all animals in data set using ssGBLUP;
4. To calculate the SNP effect: $û=\lambda DZ^{\prime }G^{*-1}{â}_{g}$;
5. To calculate the variance of each SNP:$d_{i}={û}_{i}^{2}2p_{i}\left(1-p_{i}\right)$, where I is the *i*^*th*^ marker;
6. To normalize the values of SNPs to keep constant the additive genetic variance;
7. Exit, or loop to step 2.

The effects of markers were obtained by 2 iterations from step 2 to 7. The percentage of genetic variance that is responsible for *i*^th^ region was calculated as described by Wang et al. [26]:

$$\frac{Var\left(a_{i}\right)}{\sigma_{a}^{2}}=\;\times 100=\frac{Var\left(\sum_{j=1}^{10}Z_{j}{û}_{j}\right)}{\sigma_{a}^{2}}\times 100$$

where *a*_*i*_ is the genetic value of the *i*^*th*^ region that consists of contiguous 10 consecutive SNPs, σ^2^_a_ is the total genetic variance, *Z*_*j*_ is the vector of gene content of the *j*^*th*^ SNP for all individual, and *û*_*j*_ is the marker effect of the *j*^th^ within the *i*^th^ region. The results were presented by the proportion of variance expressed by each window of 10 SNPs. In addition, the genes located at ±500 Kb of each window were considered.

### Search for genes

The chromosome segments that are responsible for more than 1.0% of additive genetic variance were selected to explore and determine possible quantitative trait loci. The bovine genome Map Viewer was used for identification of genes, available at "National Center for Biotechnology Information" (NCBI - http://www.ncbi.nlm.nih.gov) [29] in UMD3.1 version bovine genome and Ensembl Genome Browser (http://www.ensemble.org/index.html) [30]. The classification of genes for biological function, identification of metabolic pathways and enrichment of genes was performed on the website “The Database for Annotation, Visualization and Integrated Discovery (DAVID) v. 6.7” (http://david.abcc.ncifcrf.gov/) [31] and GeneCards (http://www.genecards.org/) [32].

## Results and Discussion

### Genetic parameter estimates

The (co)variance components and heritability estimates for DMI, ADG, G:F and RFI are showed in Table 2. The criteria used to diagnose the chain convergence indicated convergence of all estimated parameters. Thus, the burn-in period considered was sufficient to reach the convergence in all parameter estimates. The posterior marginal distributions of heritability estimates for feed efficiency indicator traits showed accurate values, tending to normal distribution. The symmetric distributions of central tendency statistics of analyzed traits indicated that the analyses are reliable.

**Table 2:** Heritability (h^2^) and (co)variance parameters estimate for dry matter intake (DMI), average daily gain (ADG), feed efficiency (G:F) and residual feed intake (RFI).

Trait	σ^2^_a_	σ^2^_e_	Mean h^2^	Median h^2^	SD	HPDl	HPDu
DMI	0.01	0.014	0.47	0.47	0.05	0,37	0.57
ADG	0.01	0.014	0.43	0,43	0.05	0.33	0.53
G:F	6.01x10^-5^	0.0004	0.13	0.13	0.06	0.03	0.23
RFI	0.06	0.28	0.18	0.18	0.05	0.07	0.27

σ^2^_a_ = additive genetic variance.

σ^2^_e_ = residual variance.

HPDl = lower limit for 95% of high probability density.

HPDu: upper limit for 95% of high probability density.

The heritability estimated for G:F showed low magnitude. Grion et al. [12] and Ceacero et al. [33] found low heritability for the same trait (0.17 ± 0.07, 0.14 ± 0.06, respectively). For DMI and ADG, the estimated heritability was moderate to high. Baldi et al. [34] in a study with Nellore animals and modeling the weights with random regression models, found lower heritability estimates for average daily gain during the performance test (0.21). Similar heritability estimates (0.47) to those obtained in the present study for DMI was reported by Bolormaa et al. [11] with taurine and zebu animals. The heritability obtained for RFI showed moderate magnitude. Recently, Grion et al. [12], working with Nellore animals obtained a higher RFI heritability estimate (0.33± 0.10). Bolormaa et al. [11] also reported higher heritability estimates for RFI (0.36), in a study with nine herds of *Bos Taurus*, *Bos indicus*, and crossbreeds. Also, Silva et al. [35] reported similar heritability estimates for ADG (0.39±0.08) and DMI (0.43±0.08) in a study using the same experimental population. The results of this study pointed out that there is genetic variability in selecting for feed efficiency indicator traits in Nellore cattle. Thus, it is important to known whether there are more genes involved to better understand the genetic architecture of these traits.

The known genes found in the regions that accounted for more than 1.0% of additive genetic variance are presented in tables according to the studied trait. The results indicated a total of 8, 17, 14 and 12 different windows with known genes that are responsible for more than 1.0% of the genetic variance for DMI, ADG, G:F, and RFI, respectively.

### Genomic regions

**Fig 1:**
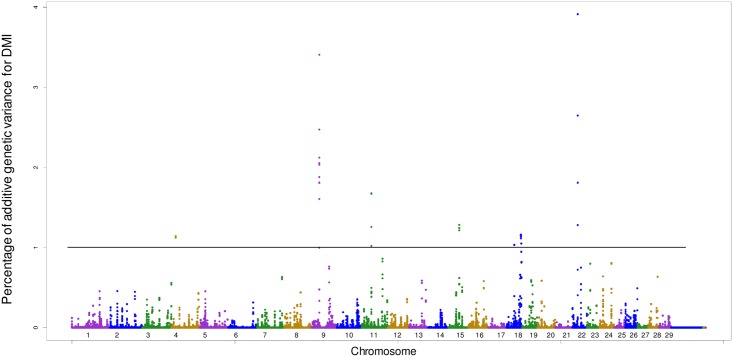
Manhattan plot of additive genetic variance expressed by windows of 10 adjacent SNPs for DMI.

For DMI, eight genomic regions that are responsible for more than 1.0% of the additive genetic variance were found (Table 3; Fig 1). The window that is responsible for the most part of additive genetic variance for DMI was located in chromosome BTA4 where one candidate gene, called *NXPH1*, was found associated with the DMI. The *RFX6*, *GPRC6A*, *FAM162B*, *KPNA5* and *ZUFSP* genes were identified in the window located in chromosome BTA9 at the 34 Mb position that is responsible for 3.40% of the additive genetic variance. The *RFX6* gene is related to the regulation of transcription. Results of a study in rats have suggested that this gene acts on the differentiation of cells in insulin production [36].

**Table 3:** Genomics regions associated with dry matter intake (DMI) in Nellore cattle, percentage of additive genetic variance and candidate genes.

Genomic region	% additive genetic variance	Candidate genes
BTA4: 18.396.753–18.454.503	1.14	***NXPH1***
BTA9: 34.179.212–34.214.753	3.40	***RFX6*, *GPRC6A*,** ***FAM162B*, *KPNA5*,** ***ZUFSP***
BTA11: 38.855.270–38.866.761	1.67	***EFEMP1*, *CCDC85A***
BTA15: 46.004.031–46.020.475	1.28	***RBMXL2*, *NLRP14*,** ***ZNF214*, *ZNF215*,** ***OR2D3*, *OR2D2*,** ***OR6A2***
BTA18: 30.155.178–30.173.492	1.03	***CDH8***
BTA18: 59.395.113–59.459.056	1.16	***LOC 100847180*,** ***LOC100336734*,** ***LOC515089*,** ***LOC787858***
BTA18: 62.231.299–62.270.553	1.04	***CACNG6*, *CACNG7*,** ***VSTM1*, *NLRP9*, *EPN1*,** ***CCDC106*, *ZNF581*,** ***ZNF580*, *ZNF784*,** ***ZNF865*, *ZNF524*, *FIZ1***
BTA22: 22.890.737–22.906.113	3.91	***LRRN1*, *CRBN*, *TRNT*,** ***IL5RA*, *CNTN4***

In the associated window located in BTA15 at the 46 Mb position the *OR2D2*, *OR2D3* and *OR10A4* genes associated with DMI were found, which have been reported to play roles of olfactory receptors, coupled to G proteins, to recognize and mediate the olfactory signals in humans and rats [37, 38]. The *ZNF214*, *ZNF215* and *NLRP14* genes were found in the same chromosome. The *NLRP14* gene encodes a protein with activity related to the immune system of the animal [39]. Three genomic regions located on chromosome BTA18 were found associated with DMI. Among the genes found in those regions, the *CACNG7* and *CACNG6* genes are associated with calcium channels [40].

The results of gene enrichment analysis and functional clusters showed that genes associated to DMI are involved in functions related to ion transport (Table A in S1 Tables). The metabolic pathways (Table 4) showed that those significant genes (p-value < 0.05) encode for a protein domain known as a zinc finger that acts as a structural element in proteins. Zinc is essential for several biochemical and cellular signaling pathways, participating in DNA and RNA synthesis and division and cell activation. Also, Zinc is essential for immune response [41].

**Table 4:** Enriched GO terms and KEGG pathways from DAVID software for DMI.

Category	Term	Count	%	p-value	*FDR (%)
INTERPRO	Zinc finger, C2H2-type	3	1.20	0.017	13.64
INTERPRO	Zinc finger, like C2H2	3	1.20	0.018	14.28
UP_SEQ_FEATURE	repeat:LRR 5	2	0.77	0.035	26.74
SMART	ZnF_C2H2	3	1.20	0.35	20.40
UP_SEQ_FEATURE	repeat:LRR 4	2	0.77	0.038	29.00
UP_SEQ_FEATURE	repeat:LRR 3	2	0.77	0.044	32.47
UP_SEQ_FEATURE	repeat:LRR 1	2	0.77	0.047	34.14
UP_SEQ_FEATURE	repeat:LRR 2	2	0.77	0.047	34.14
SP_PIR_KEYWORDS	leucine-rich repeat	2	0.77	0.080	45.78
GOTERM_MF_FAT	Metal ion binding	6	2.33	0.087	54.00
GOTERM_MF_FAT	Cation binding	6	2.33	0.091	55.60
GOTERM_MF_FAT	Ion binding	6	2.33	0.095	57.00

*FDR (%) = False Discovery Rate.

**Fig 2:**
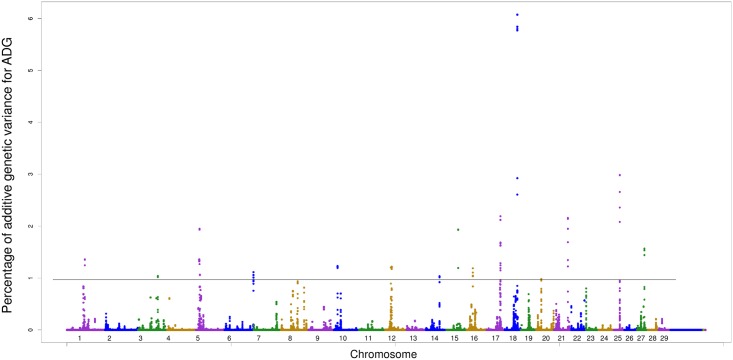
Manhattan plot of additive genetic variance expressed by windows of 10 adjacent SNPs for ADG.

For ADG, seventeen genomic regions located in 14 different chromosomes are responsible for more than 1.0% of the additive genetic variance (Table 5; Fig 2). In the window located in BTA1 at position 75 Mb, two candidate genes associated with ADG (*MB21D2* and *FGF12)* were found. The *OR52J3* and *OR51A7* genes found in a window in BTA15 encode olfactory receptors. The *CAPN8*, *CAPN2* and *TP53BP2* genes are located in BTA16, where the first two encode subunits of the calpain enzyme. Calpain is an enzyme related to the tenderness of beef after slaughter [42]. Ten genes were located in the window, in BTA18 at position 49 Mb, that are responsible for the greatest proportion of additive variance (6.07%) of ADG. Among these genes, three of them (*MAP3K10*, *CNTD2* and *AKT2*) encode kinases protein, which belongs to the largest family of proteins in eukaryotes. These genes also play an important part in intracellular communication, regulation and signal transduction, and catalyzed the phosphorylation of proteins by ATP transfer [43].

**Table 5:** Genomics regions associated with average daily gain (ADG) in Nellore cattle, percentage of additive genetic variance and candidate genes.

Genomic region	% additive genetic variance	Candidate genes
BTA1: 75.584.269–75.618.069	1.35	***MB21D2*, *FGF12***
BTA3: 85.425.169–85.443.413	1.04	***NFIA***
BTA5: 14.731.575–14.763.359	1.35	***SLC6A15*, *TSPAN19*,** ***LRRIQ1***
BTA5: 15.830.784–15.847.523	1.94	***RASSF9*, *NTS*, *MGAT4C***
BTA5: 17.563.565–17.596.734	1.06	***CEP290*, *TMTC3***
BTA6: 118.707.394–118.729.013	1.11	***CCDC96*, *TADA2B*,** ***GRPEL1*, *SORCS2*,** ***PSAPL1***
BTA10: 12.891.097–12.898.777	1.22	***SLC24A1*, *DENND4A*,** ***RAB11A*, *MEGF11*,** ***DIS3L*, *TIPIN***
BTA12: 22.039.454–22.083.431	1.20	***SLC25A15*, *MRPS31*,** ***FOXO1***
BTA12: 25.358.539–25.400.371	1.21	***CCNA1*, *SPG20*, *CCDC169*,** ***SOHLH2*, *DCLK1***
BTA14: 56.996.809–57.030.553	1.03	***KCNV1*, *SYBU*, *EBAG9*,** ***PKHD1L1*, *ENY2*, ** ***NUDCD1*, *TRHR***
BTA15: 50.448.739–50.491.730	1.93	***OR52J3*, *OR51A7***
BTA16: 27.811.695–27.823.039	1.18	***CAPN8*, *CAPN2*, *TP53BP2***
BTA17: 58.907.025–58.919.312	2.19	***SRRM4*, *SUDS3*, *TAOK3***
BTA18: 49.844.762–49.864.296	6.07	***LEUTX*, *DYRK1B*, *FBL*,** ***PSMC4*, *FCGBP*, *MAP3K10*,** ***TTC9B*, *CNTD2*, *AKT2*,** ***PLD3***
BTA21: 58.239.214–58.254.496	2.15	***LGMN*, *GOLGA5*,** ***CHGA*, *ITPK1***
BTA25: 23.226.474–23.258.055	2.98	***LCMT1*, *AQP8*, *ZKSCAN2***
BTA27: 32.802.856–32.822.191	1.56	***ZNF703*, *ERLIN2*, *PROSC*,** ***BRF2*, *RAB11FIP1*, *GOT1L1*,** ***ADRB3*, *EIF4EBP1***

The enrichment analyses for ADG revealed functional clusters related to the catabolism of macromolecules, transcription, protein kinase and binding nucleotides (Table B in S1 Tables). The gene enrichment and metabolic pathways showed that those significant genes (p <0.05) are related to endocytosis (extracellular transport) and merger of myoblasts, which are precursor cells of muscle fibers, and formation of syncytia (multinucleated cells) (Table 6).

**Table 6:** Enriched GO terms and KEGG pathways from DAVID software for ADG.

Category	Term	Count	%	p-value	*FDR (%)
GOTERM_BP_FAT	Syncytium formation by plasma membrane fusion	2	0.32	0.009	12.17
GOTERM_BP_FAT	Myoblast fusion	2	0.32	0.009	12.17
GOTERM_BP_FAT	Syncytium formation	2	0.32	0.013	15.90
SP_PIR_KEYWORDS	Activator	4	0.70	0.015	16.41
GOTERM_BP_FAT	Myotube differentiation	2	0.32	0.019	22.90
SP_PIR_KEYWORDS	Transport	8	1.30	0.036	34.11
GOTERM_MF_FAT	Transcription activator activity	3	0.50	0.042	38.72
KEGG_PATHWAY	Endocytosis	3	0.50	0.078	52.81
GOTERM_CC_FAT	Nuclear chromatin	2	0.32	0.082	60.41
GOTERM_BP _FAT	Vascular process in circulatory system	2	0.32	0.100	73.91
GOTERM_BP_FAT	Regulation of tube size	2	0.32	0.100	73.91
GOTERM_BP_FAT	Regulation	2	0.32	0.100	73.91

*FDR (%) = False Discovery Rate.

**Fig 3:**
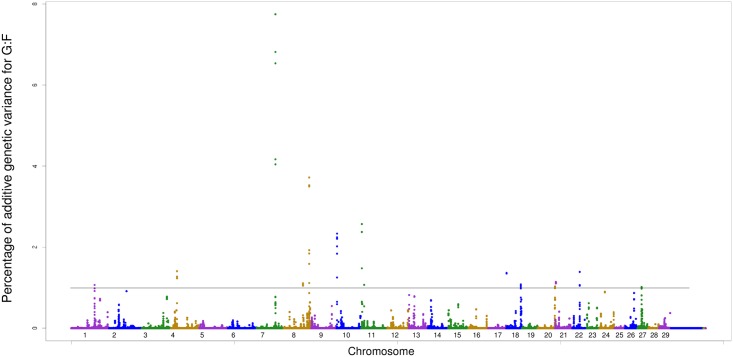
Manhattan plot of additive genetic variance expressed by windows of 10 adjacent SNPs for G:F.

Several genes were found in the 14 genomic regions that are responsible for more than 1% of the additive genetic variance of G:F (Table 7, Fig 3). The *GOLIM4* and *SERPINI1* genes were found in the window located in BTA1 at position 100 Mb. The GOLIM4 gene encodes an integral membrane protein localized in the Golgi apparatus, which is the main organelle in the secretory pathway in eukaryotic cells [44]. In chromosome BTA4 at position 26 Mb, the candidate genes *PRPS1L1* and *HDAC9* were found. The *HDAC9* gene is a histone deacetylase enzyme, which is related the transcription factor. The position 78 Mb (BTA7) made an important contribution to the additive genetic variance for G:F (7.74%), but no candidate gene was found in this genomic region. The *RASEF* and *FRMD3* genes were found in the window located in BTA8 at position 77 Mb. The *RASEF* gene encodes the GTP binding protein and binding calcium ions involved in the regulation of membrane traffic.

**Table 7:** Genomic regions associated with feed efficiency (G:F) in Nellore cattle, percentage of additive genetic variance and candidate genes.

Genomic region	% additive genetic variance	Candidate genes
BTA1: 100.014.690–100.024.850	1.06	***GOLIM4*, *SERPINI1***
BTA4: 26.970.205–27.013.467	1.40	***PRPS1L1*, *HDAC9***
BTA7: 78.617.232–78.676.806	7.74	**-**
BTA8: 77.932.482–77.966.961	1.10	***RASEF*, *FRMD3*,** ***UBQLN1*, *GKAP1***
BTA8:103.636.023–103.659.024	3.71	***SUSD1*, *PTBP3*, *HSDL2*,** ***KIAA1958*, *INIP*, *SNX30*,** ***SLC46A2***
BTA10: 2.532.364–2.549.937	2.33	***NREP*, *YTHDC2*, *KCNN2***
BTA11: 1.706.353–1.734.496	2.56	***ACOXL*, *BUB1*, *TPC3*,** ***NPHP1*, *MALL*, *MRPS5*,** ***ZNF514*, *ZNF2*, *PROM2***
BTA11: 9.544.481–9.586.492	1.07	***MRPS9*, *GPR45*,** ***TGFBRAP1*,** ***FHL2*, *TACR1*, *POLE4*, *HK2***
BTA18: 1.701.547–1.715.218	1.36	***SF3B3*, *COG4*, *FUK*,** ***ST3GAL2*, *DDX19A*, *AARS***
BTA18: 60.373.325–60.390.691	1.08	***ZNF677*, *ZNF729***
BTA20: 71.942.837–71.992.748	1.03	***CEP72*, *SLC9A3*, *EXOC3*,** ***PDCD6*, *SDHA*,** ***CCDC127*, *LRRC14B***
BTA21: 5.696.944–5.706.720	1.14	***GABRG3*, *VIMP*, *CHSY1*,** ***LRRK1*, *ALDH1A3***
BTA22: 32.257.185–32.272.017	1.38	***FRMD4B*, *LMOD3*,** ***ARL6IP5*,** ***UBA3*, *TMF1*, *EOGT***
BTA27: 17.329.309–17.350.208	1.02	-

The associated candidate genes *NREP*, *YTHDC2*, and *KCNN2* were found at the window at 2 Mb (BTA10). The *KCNN2* gene is related to the activity in calcium/potassium channels. The Ca/K channels are present in the cytosol modulate tissue concentrations regulating the potential membrane and when present in the liver it is believed that they play a role in the response to metabolic stress [45]. Nine genes were found in the window located at the position 1.7 Mb in BTA11 (*ACOXL*, *BUB1*, *TPC3*, *NPHP1*, *MALL*, *MRPS5*, *ZNF514*, *ZNF2* and *PROM2*). At the position 9 Mb in the same chromosome (BTA11), seven other candidate genes were listed: *MRPS9*, *GPR45*, *TGFBRAP1*, *FHL2*, *TACR1*, *POLE4* and *HK2*. The *GPR45* gene belongs to a family of receptors present in G proteins, and some studies in humans and mice have demonstrated that the *GPR* gene family is responsible for and is functional in the brain [46].

The *SF3B3*, *COG4*, *FUK*, *ST3GAL2*, and *AARS* genes were identified in BTA18, close to the 1700 kb region. The COG4 gene is responsible for in the Golgi apparatus, performing functions related to secretion [44]. In the same chromosome at position 60 Mb two genes were identified: *ZNF677* and *ZNF729*. Both of them encode type Zinc Fingers binding proteins, which is characterized by the coordination and stabilization of zinc ions in several processes of ion exchange [16].

Seven candidate genes for G:F were found in BTA20 at the position 71 Mb: *CEP72*, *SLC9A3*, *EXOC3*, *PDCD6*, *SDHA*, *CCDC127*, and *LRRC14B*. In humans the *SLC9A3* gene has the function of pH regulation, eliminating the acids produced by the metabolism and has proton antiporter activity, and solute carrier family 9 is involved in the exchange of sodium ions and protons, because comprises Na^+^/H^+^ exchanger proteins [47]. The window located at 5.6 Mb (BTA21) presented five candidate genes *GABRG3*, *VIMP*, *CHSY1*, *LRRK1*, and *ALDH1A3*. BTA22 presented six associated candidate genes *FRMD4B*, *LMOD3*, *ARL6IP5*, *UBA3*, *TMF1*, and *EOGT*. In BTA27 the associated genomic region did not show any gene because it is an intergenic region.

The results of gene enrichment and functional analysis reported clusters related to nucleotides, Golgi apparatus, protein transport and acetylation (Table C in S1 Tables). The metabolic pathways linked to carbohydrate metabolism, such as six-carbon polysaccharides fructose and mannose. These polysaccharides are intermediates in the glucose degradation process and glycolytic pathway, which are the main energy source for all cell types from mammals, being responsible for the ATP supply in aerobic and anaerobic conditions [48] (Table 8).

**Table 8:** Enriched GO terms and KEGG pathways from DAVID software for G:F.

Category	Term	Count	%	p-value	*FDR (%)
INTERPRO	Ribosomal protein S5 domain 2 –type fold	3	0.33	0.0055	0.60
GOTERM_CC_FAT	Golgi apparatus part	4	0.44	0.0056	5.61
GOTERM_MF_FAT	Adenyl nucleotide binding	9	1.00	0.012	12.92
GOTERM_MF_FAT	Purine nucleoside binding	9	1.00	0.013	13.58
GOTERM_MF_FAT	Nucleoside binding	9	1.00	0.013	14.00
SMART	KRAB	3	0.33	0.015	9.91
GOTERM_MF_FAT	Purine nucleotide binding	10	1.10	0.015	16.00
GOTERM_CC_FAT	Golgi apparatus	5	0.55	0.020	18.24
GOTERM_CC_FAT	Golgi membrane	3	0.33	0.025	23.00
INTERPRO	Krueppel-associated box	3	0.33	0.026	25.00
GOTERM_MF_FAT	ATP binding	8	0.90	0.029	27.32
GOTERM_MF_FAT	Adenyl ribonucleotide binding	8	0.90	0.030	28.34
GOTERM_MF_FAT	Ribonucleotide binding	9	1.00	0.035	32.42
GOTERM_MF_FAT	Purine ribonucleotide binding	9	1.00	0.035	32.42
GOTERM_MF_FAT	Nucleotide binding	10	1.10	0.043	38.43
GOTERM_MF_FAT	Solute: hydrogen antiporter activity	2	0.22	0.044	39.36
GOTERM_MF_FAT	Solute: cation antiporter activity	2	0.22	0.053	45.14
GOTERM_MF_FAT	Endomembrane system	2	0.44	0.060	45.70
KEGG_PATHWAY	Fructose and mannose metabolism	2	0.22	0.070	47.70
INTERPRO	Zinc finger, C2H2/integrase, DNA-binding	2	0.33	0.075	57.10
SMART	ZnF_C2H2	3	0.33	0.083	44.81
GOTERM_BP_FAT	Ion transport	5	0.55	0.084	65.78
GOTERM_MF_FAT	Solute: solute antiporter activity	2	0.22	0.87	63.26
GOTERM_MF_FAT	Antiporter activity	2	0.22	0.100	66.76
KEGG_PATHWAY	Amino sugar nucleotide sugar metabolism	2	0.22	0.100	59.70

*FDR (%) = False Discovery Rate.

**Fig 4:**
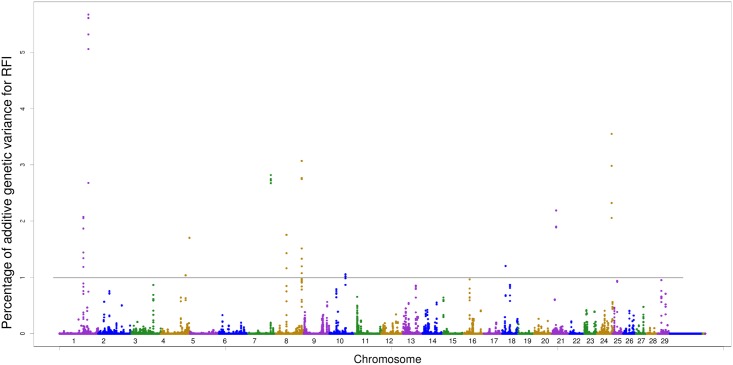
Manhattan plot of additive genetic variance expressed by windows of 10 adjacent SNPs for RFI.

A total of 12 SNP windows distributed in BTA1, BTA4, BTA7, BTA8, BTA10, BTA18, BTA21, and BTA24 are responsible for more than 1% of the additive variance for RFI (Fig 4). Mujibi et al. [49], in a study of crossbreed beef cattle, reported 11 SNP windows associated with RFI. Santana et al. [16] found two SNP windows (located in BTA8 and BTA21) associated with RFI in a Nellore cattle population using two different densities of SNP markers.

Many candidate genes identified in regions associated with RFI are coding proteins involved in the ion transportation system. This system can consume more than 10% of the total energy used by ruminants. Thus, animals with a reduced energy expenditure on this of this system can redirect energy to spend on other processes, which may influence feed efficiency [16]. In the present study, the window located at 121 Mb (BTA1) is responsible for 5.67% of the additive genetic variance for RFI. This window harbored the *ZIC1 e ZIC4* genes, which are related to ion transport, since codified protein subunits which depend on zinc ions for stability. Other genes are also related to ion transportation system, such as *GPR98*, *KCNV2* (acting on potassium channels) and *ZDHHC7*.

A total of eight candidate genes was found in BTA4, of which three of them are linked to the perception of food: *TAS2R3* and *TAS2R4*, which are related to taste through taste receptors, and *OR9A*, which encodes odor receptors. In another region of BTA4 (118 Mb) six candidates genes were found. The *LMBR1* gene, located at 118 Mb (BTA4), has the function to encode membrane proteins. In addition, other candidate genes encodes proteins with biological functions associated with many cellular processes such as transcription (*POLR3G* and *TBPL2*), cellular secretion (*KTN1* and *GOLIM4*), and transport across membranes (*TMEM178B* and *AGK*). No candidate gene was found at the position 18 Mb (BTA21) that is responsible for 2.18% of additive genetic variance for RFI (Table 9).

**Table 9:** Genomic regions associated with residual feed intake (RFI) in Nellore cattle, percentage of additive genetic variance and candidate genes.

Genomic region	% additive genetic variance	Candidate genes
BTA1: 100.014.690–100.024.850	2.07	***GOLIM4*, *SERPINI1***
BTA1: 121.639.147–121.673.712	5.67	***ZIC1*, *ZIC4***
BTA4: 105.904.240–105.916.649	1.03	***TMEM178B*, *AGK*,** ***KIAA1147*,** ***SSBP1*, *TAS2R3*, *TAS2R4*,** ***PRSS37*, *OR9A4***
BTA4: 118.565.156–118.604.811	1.70	***EN2¸CNPY1*, *RBM33*,** ***SHH*, *RNF32*, *LMBR1***
BTA7: 92.477.585–92.509.554	2.81	***POLR3G*, *LYSMD3*, *GPR98***
BTA8: 41.938.966–41.956.731	1.75	***KIAA0020*, *KCNV2***
BTA8: 103.619.063–103.646.388	3.07	***KIAA1958*, *INIP***
BTA8: 103.659.024–103.677.483	1.19	***HSDL2*, *KIAA1958*, *INIP***
BTA10: 68.372.903–68.395.669	1.05	***FBXO34*, *ATG14*,** ***TBPL2*, *KTN1***
BTA18: 11.032.341–11.066.819	1.20	***CRISPLD2*, *ZDHHC7*,** ***KIAA0513*, *FAM92B***
BTA21: 18.152.308–18.161.649	2.18	**-**
BTA24: 59.463.065–59.493.043	3.55	***CCBE1*, *PMAIP1*, *MC4R***

The results of enrichment pathway analysis indicated that genes associated with RFI (p <0.05) are related to sensory receptors that operate in food taste perception and receptors coupled to G proteins (Table 10). The enrichment by functional cluster for RFI showed that those genes are linked to cell membranes that are related to the thermodynamic equilibrium of cells. (Table D in S1 Tables). The most common mechanism to maintain the thermodynamic equilibrium of cells is ion exchange activity, maintaining cell differentiation potential through the sodium-potassium pump. For adequate pump operation, in order to maintain thermodynamic equilibrium, energy is needed (ATP to keep the sodium-potassium pump equilibrium). This process requires approximately 25% of the basal energy expenditure of an individual [50].

**Table 10:** Enriched GO terms and KEGG pathways from DAVID software for RFI.

Category	Term	Count	%	p-value	*FDR (%)
SP_PIR_KEYWORDS	G-protein coupled receptor	3	1.10	0.011	9.00
SP_PIR_KEYWORDS	Taste	2	0.73	0.012	9.85
SP_PIR_KEYWORDS	Transducer	3	1.10	0.015	12.00
INTERPRO	Mammalian taste receptor	2	0.73	0.016	12.70
GOTERM_BP_FAT	Sensory perception of taste	2	0.73	0.032	31.00
GOTERM_BP_FAT	Sensory perception of chemical stimulus	2	0.73	0.042	39.04
KEGG_PATHWAY	Taste transduction	2	0.73	0.045	27.84
SP_PIR_KEYWORDS	Sensory transduction	2	0.73	0.054	37.63
GOTERM_BP_FAT	G-protein coupled receptor protein signaling pathway	5	1.83	0.062	52.00
SP_PIR_KEYWORDS	Receptor	3	1.10	0.073	47.28

*FDR (%) = False Discovery Rate.

The *GOLIM4* and *SERPINI1* genes, located in BTA1, have been linked to more than one trait, such as G:F and RFI. It was observed that few SNP windows located nearby in the same chromosome were associated with more than one trait like in BTA8, where two SNP windows were associated with RFI and G:F. Seven genes related the zinc finger protein domain located in BTA18 were found in several nearby SNP windows associated with G:F and DMI. These results could be due to pleitropic effects, which means that the expression of different traits could be influenced by the same set of genes which acts in a coordinated manner to contribute to feed efficiency.

It is important to highlight that the results obtained in this study are also supported by previous studies. Rolf et al. [14] working with an Angus population, reported genomic regions associated with DMI, RFI and ADG close to those obtained in this study for the same traits. Bolormaa et al. [11] reported seven genomic regions near (at a maximum distance of 3 Mb) those identified in this study for RFI. Recently, Karisa et al. [51] reported a candidate gene (*CYP2B*) associated with RFI in BTA18 (49 Mb), at the same position that the present study identified candidate genes for ADG. Recently, in a study with a Nellore cattle population, De Oliveira et al. [52] reported differently located candidate genes for feed efficiency traits than found in this study (*HRH4*, *ALDH7A1*, *APOA2*, *LIN7C*, *CXADR*, *ADAM12* and *MAP7*). However, the genes described in that study have similar gene ontology (immune system, energy and ion metabolism) to the genes reported in the present study.

The large number of genomic regions associated with feed efficiency traits obtained in this study should support a better understanding the genetic and physiological mechanisms that determine growth, feed intake and feed efficiency in zebu animals. The results demonstrate the probability that these traits have their expression controlled by many QTL with small individual effects, confirming their polygenic nature. The identification of relevant genes might be a difficult task, since the additive genetic variance contribution from each region or SNP window for many traits was lower than expected. Thus, strategies such as genomic selection that take into consideration the variability among all markers might be a more adequate alternative to improve these traits.

In recent years there has been growing concern about the contribution of the beef industry to climate change. Livestock in particular has been identified as a major contributor to global warming according to the FAO report [53]. Climate change and variability impacts on livestock productivity, and especially on the economic and political behavior of international markets, constituting the main current threat to beef exports in many countries. The ability to become a supplier country of reliable and safe food to the world should be combined with environmental sustainability. The results obtained in this study show that it is possible to improve beef cattle feed efficiency, trough selection using genomic information, and reach additional benefits to the environment by reducing greenhouse gas emissions. Finally, the data set used in this study belongs to a beef cattle research farm, which provides selected sires to commercial herds in many regions of Brazil. Therefore, the information found in this study will contribute to the selection for animals with better feed efficiency and increased environmental and social sustainability.

## Conclusion

The results of this study pointed out that selection for feed efficiency indicator traits is feasible in Nellore cattle under tropical conditions. It is expected that the response to selection would be higher for RFI than for G:F. Several genomic regions harboring possible QTL for feed efficiency indicator traits were identified. The candidate genes identified are involved in energy and protein metabolism, ion transport, transmembrane transport, the olfactory system, the immune system, secretion (Golgi apparatus) and cellular activity (cell multiplication). The identification of these regions and their respective candidate genes should contribute to the formation of a genetic basis for Nellore feed efficiency indicator traits, and these results would support the selection for these traits.

## Supporting Information

S1 Tables Table A—Functional cluster enrichment analysis via DAVID database for DMI.
Table B—Functional cluster enrichment analysis via DAVID database for ADG.
Table C—Functional cluster enrichment analysis via DAVID database for G:F.
Table D—Functional cluster enrichment analysis via DAVID database for RFI.
(DOCX)
