# Correction: Genomic Regions Associated with Feed Efficiency Indicator Traits in an Experimental Nellore Cattle Population

**DOI:** 10.1371/journal.pone.0171845

**Published:** 2017-02-06

**Authors:** Bianca Ferreira Olivieri, Maria Eugênia Zerlotti Mercadante, Joslaine Noely dos Santos Gonçalves Cyrillo, Renata Helena Branco, Sarah Figueiredo Martins Bonilha, Lucia Galvão de Albuquerque, Rafael Medeiros de Oliveira Silva, Fernando Baldi

The following information is missing from the Funding section: This study was supported by Sao Paulo Research Foundation (FAPESP, grant #2011/21241-0).
