# Supplementary material for: Genomic Regions Associated with Feed Efficiency Indicator Traits in an Experimental Nellore Cattle Population
Source: PLoS One. 2016 Oct 19;11(10):e0164390. doi: 10.1371/journal.pone.0164390 (PMC5070821; doi:10.1371/journal.pone.0164390)
Supplement: S1 Tables — Table A—Functional cluster enrichment analysis via DAVID database for DMI. Table B—Functional cluster enrichment analysis via DAVID database for ADG. Table C—Functional cluster enrichment analysis via DAVID database for G:F. Table D—Functional cluster enrichment analysis via DAVID database for RFI. (DOCX) [file pone.0164390.s001.docx]

**S1 Table A**. - Functional cluster enrichment analysis via DAVID database for dry matter intake.

| **Annotation Cluster 1** | **Enrichment Score: 0,8599030396024786** | | | | |
| --- | --- | --- | --- | --- | --- |
| **Categoria** | **Termo** | **Count** | **%** | **p-value** | **FDR** |
| INTERPRO | Zinc finger, C2H2-type | 3 | 15.79 | 0.0172 | 13.64 |
| INTERPRO | Zinc finger, C2H2-like | 3 | 15.79 | 0.0181 | 14.29 |
| SMART | ZnF_C2H2 | 3 | 15.79 | 0.0265 | 14.87 |
| GOTERM_MF_FAT | zinc ion binding | 3 | 15.79 | 0.3929 | 98.76 |
| GOTERM_MF_FAT | metal ion binding | 4 | 21.05 | 0.4220 | 99.19 |
| GOTERM_MF_FAT | cation binding | 4 | 21.05 | 0.4305 | 99.29 |
| GOTERM_MF_FAT | ion binding | 4 | 21.05 | 0.4377 | 99.37 |
| GOTERM_MF_FAT | transition metal ion binding | 3 | 15.79 | 0.5128 | 99.82 |

**S1 Table B. -** Functional cluster enrichment analysis via DAVID database for average daily gain.

| **Annotation Cluster 1** | **Enrichment Score: 0,9325203566278826** |  |  |  |  |
| --- | --- | --- | --- | --- | --- |
| **Category** | **Term** | **Count** | **%** | **p-value** | **FDR** |
| GOTERM_BP_FAT | negative regulation of macromolecule biosynthetic process | 3 | 5.882 | 0.104 | 76.11 |
| GOTERM_BP_FAT | negative regulation of cellular biosynthetic process | 3 | 5.882 | 0.107 | 77.07 |
| GOTERM_BP_FAT | negative regulation of biosynthetic process | 3 | 5.882 | 0.113 | 78.91 |
| GOTERM_BP_FAT | negative regulation of macromolecule metabolic process | 3 | 5.882 | 0.148 | 87.67 |
|  |  |  |  |  |  |
| **Annotation Cluster 2** | **Enrichment Score: 0,6333930920755426** |  |  |  |  |
| **Category** | **Term** | **Count** | **%** | **p-value** | **FDR** |
| SP_PIR_KEYWORDS | activator | 4 | 7.84 | 0.015 | 15.10 |
| GOTERM_MF_FAT | transcription activator activity | 3 | 5.88 | 0.037 | 34.71 |
| GOTERM_MF_FAT | transcription regulator activity | 5 | 9.80 | 0.137 | 80.90 |
| GOTERM_CC_FAT | nucleoplasm part | 3 | 5.88 | 0.171 | 86.60 |
| GOTERM_BP_FAT | transcription | 4 | 7.84 | 0.176 | 91.96 |
| GOTERM_CC_FAT | nucleoplasm | 3 | 5.88 | 0.221 | 93.06 |
| SP_PIR_KEYWORDS | transcription regulation | 4 | 7.84 | 0.245 | 95.59 |
| **Category** | **Term** | **Count** | **%** | **p-value** | **FDR** |
| GOTERM_CC_FAT | intracellular organelle lumen | 4 | 7.84 | 0.298 | 97.72 |
| GOTERM_CC_FAT | organelle lumen | 4 | 7.84 | 0.299 | 97.75 |
| GOTERM_CC_FAT | membrane-enclosed lumen | 4 | 7.84 | 0.321 | 98.41 |
| SP_PIR_KEYWORDS | Transcription | 4 | 7.84 | 0.322 | 98.67 |
| GOTERM_CC_FAT | nuclear lumen | 3 | 5.88 | 0.415 | 99.67 |
| GOTERM_BP_FAT | regulation of transcription | 5 | 9.80 | 0.460 | 99.97 |
| SP_PIR_KEYWORDS | nucleus | 6 | 11.76 | 0.638 | 100.00 |
| GOTERM_BP_FAT | regulation of transcription, DNA-dependent | 3 | 5.88 | 0.729 | 100.00 |
| GOTERM_BP_FAT | regulation of RNA metabolic process | 3 | 5.88 | 0.738 | 100.00 |
|  |  |  |  |  |  |
| **Annotation Cluster 3** | **Enrichment Score: 0,5012340744445127** |  |  |  |  |
| **Category** | **Term** | **Count** | **%** | **p-value** | **FDR** |
| GOTERM_MF_FAT | purine nucleotide binding | 7 | 13.73 | 0.174 | 88.36 |
| GOTERM_MF_FAT | adenyl nucleotide binding | 6 | 11.76 | 0.181 | 89.45 |
| GOTERM_MF_FAT | purine nucleoside binding | 6 | 11.76 | 0.186 | 90.14 |
| GOTERM_MF_FAT | nucleoside binding | 6 | 11.76 | 0.189 | 90.54 |
| **Category** | **Term** | **Count** | **%** | **p-value** | **FDR** |
| GOTERM_MF_FAT | nucleotide binding | 7 | 13.73 | 0.295 | 98.04 |
| GOTERM_MF_FAT | purine ribonucleotide binding | 6 | 11.76 | 0.301 | 98.20 |
| GOTERM_MF_FAT | ribonucleotide binding | 6 | 11.76 | 0.301 | 98.20 |
| SP_PIR_KEYWORDS | kinase | 3 | 5.88 | 0.310 | 98.38 |
| GOTERM_MF_FAT | ATP binding | 5 | 9.80 | 0.320 | 98.68 |
| GOTERM_MF_FAT | adenyl ribonucleotide binding | 5 | 9.80 | 0.326 | 98.81 |
| GOTERM_MF_FAT | protein kinase activity | 3 | 5.88 | 0.335 | 98.98 |
| GOTERM_BP_FAT | protein amino acid phosphorylation | 3 | 5.88 | 0.385 | 99.82 |
| SP_PIR_KEYWORDS | nucleotide-binding | 5 | 9.80 | 0.395 | 99.63 |
| SP_PIR_KEYWORDS | ATP-binding | 4 | 7.84 | 0.397 | 99.64 |
| GOTERM_BP_FAT | phosphorylation | 3 | 5.88 | 0.462 | 99.97 |
| GOTERM_BP_FAT | phosphorus metabolic process | 3 | 5.88 | 0.580 | 100.00 |
| GOTERM_BP_FAT | phosphate metabolic process | 3 | 5.88 | 0.580 | 100.00 |
|  |  |  |  |  |  |
| **Annotation Cluster 4** | **Enrichment Score: 0,45376291155988574** |  |  |  |  |
| **Category** | **Term** | **Count** | **%** | **p-value** | **FDR** |
| GOTERM_BP_FAT | protein transport | 3 | 5.88 | 0.336 | 99.52 |
| GOTERM_BP_FAT | establishment of protein localization | 3 | 5.88 | 0.338 | 99.53 |
| GOTERM_BP_FAT | protein localization | 3 | 5.88 | 0.383 | 99.82 |
|  |  |  |  |  |  |
| **Annotation Cluster 5** | **Enrichment Score: 0,451237654137822** |  |  |  |  |
| **Category** | **Term** | **Count** | **%** | **p-value** | **FDR** |
| UP_SEQ_FEATURE | topological domain:Cytoplasmic | 7 | 13.73 | 0.099 | 68.17 |
| SP_PIR_KEYWORDS | cell membrane | 5 | 9.80 | 0.137 | 80.56 |
| SP_PIR_KEYWORDS | glycoprotein | 7 | 13.73 | 0.275 | 97.21 |
| GOTERM_CC_FAT | plasma membrane | 6 | 11.76 | 0.352 | 99.04 |
| SP_PIR_KEYWORDS | transmembrane | 9 | 17.65 | 0.386 | 99.56 |
| UP_SEQ_FEATURE | topological domain:Extracellular | 4 | 7.84 | 0.418 | 99.74 |
| UP_SEQ_FEATURE | glycosylation site:N-linked (GlcNAc,,,) | 6 | 11.76 | 0.427 | 99.77 |
| SP_PIR_KEYWORDS | membrane | 10 | 19.61 | 0.452 | 99.88 |
| UP_SEQ_FEATURE | transmembrane region | 7 | 13.73 | 0.478 | 99.92 |
| **Category** | **Term** | **Count** | **%** | **p-value** | **FDR** |
| GOTERM_CC_FAT | integral to membrane | 10 | 19.61 | 0.723 | 100.00 |
| GOTERM_CC_FAT | intrinsic to membrane | 10 | 19.61 | 0.766 | 100.00 |
|  |  |  |  |  |  |
| **Annotation Cluster 6** | **Enrichment Score: 0,2459677891883038** |  |  |  |  |
| **Category** | **Term** | **Count** | **%** | **p-value** | **FDR** |
| SP_PIR_KEYWORDS | calcium | 3 | 5.88 | 0.375 | 99.47 |
| GOTERM_MF_FAT | calcium ion binding | 3 | 5.88 | 0.442 | 99.86 |
| GOTERM_MF_FAT | metal ion binding | 7 | 13.73 | 0.697 | 100.00 |
| GOTERM_MF_FAT | cation binding | 7 | 13.73 | 0.709 | 100.00 |
| GOTERM_MF_FAT | ion binding | 7 | 13.73 | 0.719 | 100.00 |
|  |  |  |  |  |  |
| **Annotation Cluster 7** | **Enrichment Score: 0,22734908854405084** |  |  |  |  |
| **Category** | **Term** | **Count** | **%** | **p-value** | **FDR** |
| SP_PIR_KEYWORDS | glycoprotein | 7 | 13.73 | 0.275 | 97.21 |
| SP_PIR_KEYWORDS | disulfide bond | 3 | 5.88 | 0.867 | 100.00 |
| UP_SEQ_FEATURE | disulfide bond | 3 | 5.88 | 0.872 | 100.00 |

**S1 Table C.** - Functional cluster enrichment analysis via DAVID database for feed efficiency.

| **Annotation Cluster 1** | **Enrichment Score: 1,5344117928688148** | | | | |
| --- | --- | --- | --- | --- | --- |
| **Category** | **Term** | **Count** | **%** | **p-value** | **FDR** |
| GOTERM_MF_FAT | ATP binding | 8 | 18.60 | 0.015 | 15.59 |
| GOTERM_MF_FAT | adenyl ribonucleotide binding | 8 | 18.60 | 0.016 | 16.26 |
| GOTERM_MF_FAT | purine ribonucleotide binding | 9 | 20.93 | 0.017 | 17.75 |
| GOTERM_MF_FAT | ribonucleotide binding | 9 | 20.93 | 0.017 | 17.75 |
| GOTERM_MF_FAT | adenyl nucleotide binding | 8 | 18.60 | 0.021 | 20.81 |
| GOTERM_MF_FAT | purine nucleoside binding | 8 | 18.60 | 0.022 | 21.69 |
| GOTERM_MF_FAT | purine nucleotide binding | 9 | 20.93 | 0.022 | 22.22 |
| GOTERM_MF_FAT | nucleoside binding | 8 | 18.60 | 0.022 | 22.22 |
| GOTERM_MF_FAT | nucleotide binding | 9 | 20.93 | 0.056 | 46.75 |
| SP_PIR_KEYWORDS | Nucleotide binding | 4 | 9.30 | 0.396 | 99.24 |
|  |  |  |  |  |  |
| **Annotation Cluster 2** | **Enrichment Score: 1,2910980583495473** | | |  |  |
| **Category** | **Term** | **Count** | **%** | **p-value** | **FDR** |
| GOTERM_CC_FAT | Golgi apparatus part | 4 | 9.30 | 0.004 | 4.094 |
| GOTERM_CC_FAT | Golgi apparatus | 5 | 11.63 | 0.013 | 12.69 |
| **Category** | **Term** | **Count** | **%** | **p-value** | **FDR** |
| GOTERM_CC_FAT | Golgi membrane | 3 | 6.98 | 0.02 | 18.67 |
| GOTERM_CC_FAT | endomembrane system | 4 | 9.30 | 0.044 | 36.55 |
| GOTERM_CC_FAT | organelle membrane | 3 | 6.98 | 0.414 | 99.54 |
| SP_PIR_KEYWORDS | membrane | 5 | 11.63 | 0.877 | 99.99 |
|  |  |  |  |  |  |
| **Annotation Cluster 3** | **Enrichment Score: 0,6842922085243253** | | | |  |
| **Category** | **Term** | **Count** | **%** | **p-value** | **FDR** |
| GOTERM_BP_FAT | protein transport | 4 | 9.30 | 0.114 | 76.54 |
| GOTERM_BP_FAT | establishment of protein localization | 4 | 9.30 | 0.115 | 76.74 |
| SP_PIR_KEYWORDS | protein transport | 3 | 6.98 | 0.124 | 72.32 |
| GOTERM_BP_FAT | protein localization | 4 | 9.30 | 0.143 | 84.13 |
| SP_PIR_KEYWORDS | acetylation | 5 | 11.63 | 0.503 | 99.88 |
| SP_PIR_KEYWORDS | transport | 3 | 6.98 | 0.665 | 99.99 |
|  |  |  |  |  |  |
| **Annotation Cluster 4** | **Enrichment Score: 0,6688911260597424** | | |  |  |
| **Category** | **Term** | **Count** | **%** | **p-value** | **FDR** |
| SMART | KRAB | 3 | 6.98 | 0.013 | 8.26 |
| INTERPRO | Krueppel-associated box | 3 | 6.98 | 0.023 | 21.82 |
| **Category** | **Term** | **Count** | **%** | **p-value** | **FDR** |
| INTERPRO | Zinc finger, C2H2-type/integrase, DNA-binding | 3 | 6.98 | 0.067 | 52.09 |
| SMART | ZnF_C2H2 | 3 | 6.98 | 0.071 | 39.17 |
| INTERPRO | Zinc finger, C2H2-type | 3 | 6.98 | 0.114 | 72.37 |
| INTERPRO | Zinc finger, C2H2-like | 3 | 6.98 | 0.119 | 73.98 |
| SP_PIR_KEYWORDS | nucleus | 7 | 16.28 | 0.164 | 82.36 |
| SP_PIR_KEYWORDS | Transcription | 4 | 9.30 | 0.165 | 82.56 |
| GOTERM_BP_FAT | transcription | 4 | 9.30 | 0.176 | 90.06 |
| SP_PIR_KEYWORDS | zinc | 4 | 9.30 | 0.325 | 97.75 |
| SP_PIR_KEYWORDS | metal-binding | 5 | 11.63 | 0.358 | 98.62 |
| GOTERM_BP_FAT | regulation of transcription | 5 | 11.63 | 0.460 | 99.93 |
| GOTERM_BP_FAT | regulation of transcription, DNA-dependent | 4 | 9.30 | 0.463 | 99.93 |
| GOTERM_BP_FAT | regulation of RNA metabolic process | 4 | 9.30 | 0.475 | 99.95 |
| GOTERM_MF_FAT | metal ion binding | 7 | 16.28 | 0.657 | 99.99 |
| GOTERM_MF_FAT | cation binding | 7 | 16.28 | 0.669 | 99.99 |
| GOTERM_MF_FAT | ion binding | 7 | 16.28 | 0.680 | 99.99 |
| GOTERM_MF_FAT | zinc ion binding | 4 | 9.30 | 0.737 | 99.99 |
| GOTERM_MF_FAT | transition metal ion binding | 4 | 9.30 | 0.864 | 99.99 |
|  |  |  |  |  |  |
| **Annotation Cluster 5** | **Enrichment Score: 0,31684937415727393** | | |  |  |
| **Category** | **Term** | **Count** | **%** | **p-value** | **FDR** |
| GOTERM_BP_FAT | ion transport | 4 | 9.30 | 0.190 | 91.92 |
| GOTERM_CC_FAT | integral to membrane | 9 | 20.93 | 0.546 | 99.96 |
| GOTERM_CC_FAT | intrinsic to membrane | 9 | 20.93 | 0.593 | 99.98 |
| SP_PIR_KEYWORDS | membrane | 5 | 11.63 | 0.878 | 99.99 |
|  |  |  |  |  |  |
| **Annotation Cluster 6** | **Enrichment Score: 0,269641669383175** | | | |  |
| **Category** | **Term** | **Count** | **%** | **p-value** | **FDR** |
| GOTERM_BP_FAT | phosphorylation | 3 | 6.98 | 0.462 | 99.99 |
| GOTERM_BP_FAT | phosphorus metabolic process | 3 | 6.98 | 0.580 | 99.99 |
| GOTERM_BP_FAT | phosphate metabolic process | 3 | 6.98 | 0.580 | 99.99 |

**S1 Table D.** - Functional cluster enrichment analysis via DAVID database for residual feed intake.

| **Annotation Cluster 1** | **Enrichment Score: 0,9695587223163133** | | | | |
| --- | --- | --- | --- | --- | --- |
| **Category** | **Term** | **Count** | **%** | **p-value** | **FDR** |
| SP_PIR_KEYWORDS | G-protein coupled receptor | 3 | 13.04 | 0.008 | 6.40 |
| SP_PIR_KEYWORDS | transducer | 3 | 13.04 | 0.011 | 8.58 |
| GOTERM_BP_FAT | G-protein coupled receptor protein signaling pathway | 5 | 21.74 | 0.047 | 40.53 |
| SP_PIR_KEYWORDS | receptor | 3 | 13.04 | 0.055 | 36.81 |
| GOTERM_BP_FAT | cell surface receptor linked signal transduction | 5 | 21.74 | 0.093 | 65.30 |
| SP_PIR_KEYWORDS | transmembrane | 3 | 13.04 | 0.401 | 98.44 |
| SP_PIR_KEYWORDS | membrane | 3 | 13.04 | 0.506 | 99.67 |
| GOTERM_CC_FAT | integral to membrane | 4 | 17.39 | 0.647 | 99.97 |
| GOTERM_CC_FAT | intrinsic to membrane | 4 | 17.39 | 0.674 | 99.98 |
|  |  |  |  |  |  |
|  |  |  |  |  |  |
| **Annotation Cluster 2** | **Enrichment Score: 0,1394713957286039** | | | | |
| **Category** | **Term** | **Count** | **%** | **p-value** | **FDR** |
| GOTERM_MF_FAT | metal ion binding | 3 | 13.04 | 0.719 | 100.00 |
|  |  |  |  |  |  |
| **Category** | **Term** | **Count** | **%** | **p-value** | **FDR** |
| GOTERM_MF_FAT | cation binding | 3 | 13.04 | 0.726 | 100.00 |
| GOTERM_MF_FAT | ion binding | 3 | 13.04 | 0.732 | 100.00 |
